# Glucocorticoid Stress Responses of Reintroduced Tigers in Relation to Anthropogenic Disturbance in Sariska Tiger Reserve in India

**DOI:** 10.1371/journal.pone.0127626

**Published:** 2015-06-10

**Authors:** Subhadeep Bhattacharjee, Vinod Kumar, Mithileshwari Chandrasekhar, Manjari Malviya, Andre Ganswindt, Krishnamurthy Ramesh, Kalyanasundaram Sankar, Govindhaswamy Umapathy

**Affiliations:** 1 Wildlife Institute of India, Chandrabani, Dehradun, Uttarakhand, India; 2 Laboratory for the Conservation of Endangered Species (LaCONES), CSIR-Centre for Cellular and Molecular Biology (CCMB), Hyderabad, India; 3 Endocrine Research Laboratory, Department of Anatomy and Physiology, University of Pretoria, Onderstepoort, 0110, South Africa; University of Hyderabad, INDIA

## Abstract

Tiger (*Panthera tigris*), an endangered species, is under severe threat from poaching, habitat loss, prey depletion and habitat disturbance. Such factors have been reported causing local extermination of tiger populations including in one of the most important reserves in India, namely Sariska Tiger Reserve (STR) in northwestern India. Consequently, tigers were reintroduced in STR between 2008 and 2010, but inadequate breeding success was observed over the years, thus invoking an investigation to ascertain physiological correlates. In the present study, we report glucocorticoid stress responses of the reintroduced tigers in relation to anthropogenic disturbance in the STR from 2011 to 2013. We found anthropogenic disturbance such as encounter rates of livestock and humans, distance to roads and efforts to kill domestic livestock associated with an elevation in fecal glucocorticoid metabolite (fGCM) concentrations in the monitored tigers. In this regard, female tigers seem more sensitive to such disturbance than males. It was possible to discern that tiger’s fGCM levels were significantly positively related to the time spent in disturbed areas. Resulting management recommendations include relocation of villages from core areas and restriction of all anthropogenic activities in the entire STR.

## Introduction

Reintroduction of any species to its former geographic range, where it was locally exterminated, is the last resort for conservation biologists and wildlife managers interested in species recovery programs. Of the various species, reintroduction of carnivores is considered to be a more effective step towards conservation and restoring integrity of natural ecosystems [1] because large carnivores maintain ecological balance as apex predators in e.g. forest areas [2]. However, reintroduction of carnivores in its original habitat is more challenging both biologically and politically [3], especially large carnivores require extensive habitat which often result in human-animal conflicts [3,4]. In the past, many carnivores have been reintroduced such as the Eurasian lynx (*Lynx lynx*) to the Alp mountain range in Europe [5], the Canadian lynx (*Lynx canadensis*) to Colorado in the US [6], the gray wolf (*Canis lupus*) to the Yellowstone National Park in the US [7], or the black-footed ferret (*Mustela nigripes*) to the central prairies of the United States [8]. The success of a reintroduction program depends on a variety of key factors including the biology of the species, number of founders, environmental variation, genetic variability, intra-specific competition and reproductive success [9,10]. So far, most of the reintroduction programs failed due to poorly conceptualized planning and subsequent monitoring, resulting in viable populations in only 11% of the cases [11,12]. It therefore becomes highly imperative that reintroduction programs should be based on sound scientific principles and methodology to increase their probability of success. In contrast, the successful reintroduction of the African lion (*Panthera leo*) and African wild dog (*Lycaon pictus*) in East Africa during 1996 and 2001 are just two of the few examples that enriched our knowledge regarding the science and management involved during carnivore reintroduction processes [13].

The tiger (*Panthera tigris*) is an endangered species, restricted to less than 0.6% of its historical range and the remaining estimated global populations of 3000–3500 individuals are under severe threat due to poaching, habitat loss and prey depletion [14–16]. India is one of the important countries for tiger conservation, as it supports ca. 50% of the global wild tiger populations [17]. It is therefore one of the key players for global tiger recovery [18]. Although intense tiger conservation efforts have been made through “Project Tiger” in India since 1972, their population continue to be threatened due to poaching, habitat loss and prey depletion in many parts of India, even resulting in local extinction in protected areas namely Sariska in Western India and Panna in central India in the recent past. As part of the species recovery and conservation management program, tigers were reintroduced into Sariska and Panna Tiger Reserves during 2008–2010 and 2009–2013 respectively [19]. Reintroduced tigers in Panna Tiger Reserve (*n* = 6; 4 females, 2 males) have been successful in breeding and have given births regularly [20]. In contrast, the reintroduced tigers in Sariska (*n* = 5; 3 females, 2 males) did not show breeding success until recently, June 2012 [21].

The role of stress in population decline is not clearly understood and might be multifaceted. Glucocorticoids (cortisol and corticosterone) are released under stressful conditions to help the organism defend itself against a perceived stressor [22]. Although the short term release of these hormones is known to increase fitness via energy mobilization, prolonged periods of high glucocorticoid concentrations are known to decrease fitness by affecting reproductive success, immuno-suppression, growth, and muscular atrophy [23–26]. Long-term anthropogenic disturbance has been shown to increase glucocorticoid levels in many wildlife taxa, e.g. amphibians [27], reptiles [28], birds [29] and mammals [30–32]). As human induced stress can directly affect health and reproduction in wildlife [33–35], it might lead to an overall population decline [36], especially in species which are already facing threats of extinction due to other factors such as poaching, decline of prey and quality of habitat.

Although stress-related hormones can be measured in various biological matrices, non-invasive methods using faeces as hormone matrix, have gained popularity as a more practical approach, especially in free-roaming animals [37]. It allows researchers to link the endocrine status of animals to behavior or other life-history traits without interfering with the natural behavior of the animal due to capture or restraint for invasive sampling [38].

**Fig 1:**
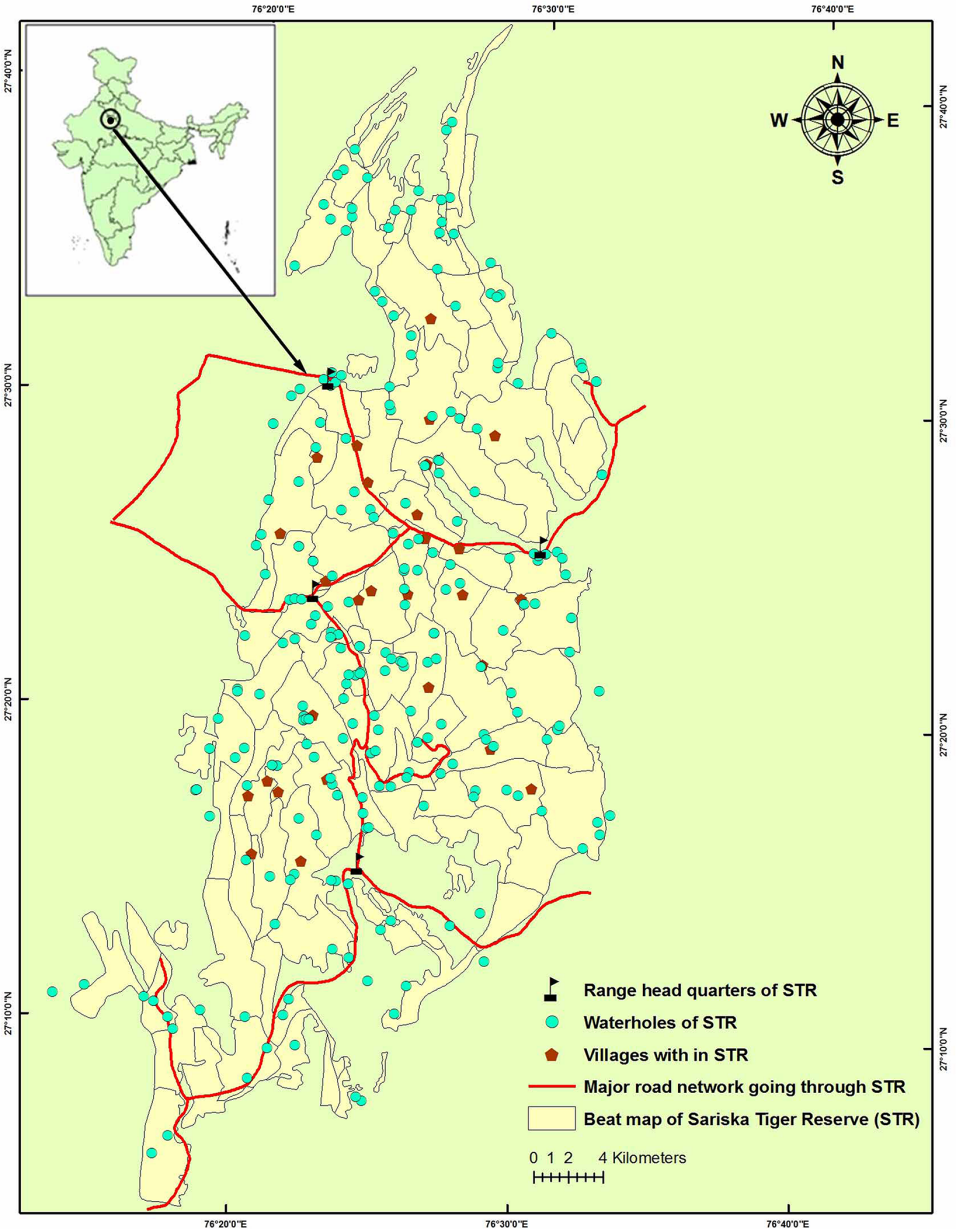
The study area, Sariska Tiger Reserve, Rajasthan, India.

About 10000 people from 32 villages along with 19132 livestock[39], live inside the Sariska Tiger Reserve (STR), which seems a serious threat to the survival of tigers [21] (Fig 1). These people are traditionally pastoralist communities who totally depend on forests for their livelihood [21]. Sankar et al [40] reported that livestock moved on an average of 3.3 km around each village, thus leaving only 15% of park without livestock presence and anthropogenic activities. These livestock formed about 19% of overall prey of tigers in Sariska although only the unguarded livestock away from the villages were killed by tigers [21]. Further, two major state highways, the Alwar-Thanagazhi-Jaipur and the Sariska-Kalighati-Tehla, which are in total about 44 km long, traverse through the center of the National Park. The presence of a large number of people, livestock and busy road inside the park would certainly have an impact on the entire wildlife and the ecosystem. With this background, it was hypothesized that stress caused by these anthropogenic pressures in the habitat might have an influence on the reproductive potential of the introduced tigers in Sariska. Therefore, the present study was aimed to assess adrenocortical activity as a measure of stress in these reintroduced tigers with reference to anthropogenic variables which might give us some insight about the origin of stress and also help us in providing further management recommendations for policy implementation to ensure a sustainable future for the reintroduced tigers in STR.

## Materials and Methods

### Study Area

#### Sariska Tiger Reserve

The Sariska Tiger Reserve (STR), (76°17ˈ E to 76°34ˈE and 27°5ˈ to 27°33ˈ N) is situated in the mountain range of the Aravallis and lies in the semi-arid part of Rajasthan [41]. It became a wildlife sanctuary in 1955 and Tiger Reserve in 1979. The total area of the Tiger Reserve is 881 km^2^ along with recently notified (in July 2012) buffer zone of 334 km^2^. Therefore, the effective total area of Sariska Tiger Reserve after July 2012 is 1203.63 km^2^. The terrain is undulating to hilly in nature and has numerous large (Sariska—Kalighati and Umri etc.) to narrow valleys, two large plateaus—Kiraska and Kankwari and two large lakes, Mansarovar and Somasagar. The altitude of Sariska ranges from 240 to 777 m. The vegetation of Sariska corresponds to northern tropical dry deciduous forests and northern tropical thorn forest [42]. Apart from the five reintroduced tigers and two new born cubs (no resident tigers), other carnivores present in STR are leopard *(Panthera pardus)*, striped hyaena *(Hyaena hyaena)*, jackal *(Canis aureus)*, jungle cat (*Felis chaus*), common mongoose *(Herpestes edwardsi*), small Indian mongoose (*H*. *auropunctatus*), ruddy mongoose (*H*. *smithi*), palm civet (*Paradoxurus hermaphroditus*), small Indian civet (*Viverricula indica*) and ratel (*Mellivora camensis*). Chital (*Axis axis*), sambar (*Rusa unicolor*), nilgai (*Boselaphus tragocamelus*) and wild pig (*Sus scrofa*) which are the major prey species for tigers found in Sariska. Other wild prey species found are common langur (*Semnopethicus entellus*), Rhesus macaque (*Macaca mulatta*), porcupine (*Hystrix indica*), rufous tailed hare (*Lepus nigricollis ruficaudatus*), and Indian peafowl (*Pavo cristatus*). There are 32 villages located inside the Tiger Reserve, out of which ten are in the Sariska notified National Park area. These ten villages in the notified National Park are due for relocation since 1984. Two villages Umri and Rotkala have been relocated from STR during 2012–2013.

### Monitoring tigers, scat collection and preservation

Five tigers at Sariska Tiger Reserve (ST2 female, ST3 female, ST4 male, ST5 female and ST6 male) were fitted with VHF-GPS-Argos radio transmitters (from Telonics Inc., Arizona, USA) between July 2008 and February 2011 and subsequently tracked on a daily basis. Permissions for capturing and radiocollaring of tigers were given by the Chief Wildlife Warden, Rajasthan Forest Department, Rajasthan and the National Tiger Conservation Authority, Ministry of Environment and Forests, Government of India (Reference letters No. 2(3)/2005-PT dated 4.4.2008 and No. 2(3)/2005-PT dated 15.4.2008). Three to four radio locations of each animal were obtained every day by periodic monitoring at an interval of four to six hours using “Homing in” and “Triangulation” techniques from three to four known reference points [43] and also by satellite tracking of Argos satellites (www.argos-system.org; Ramonville Saint-Agne, France). All handling protocols were performed in accordance with the guidelines of National Tiger Conservation Authority, Ministry of Environment and Forests, Government of India. In addition the handling and other procedures were also approved by the Institutional Animal Ethics Committee of Wildlife Institute of India.

We collected visually-appearing fresh samples (approx. 1 day old based on the outline shape and wetness) from the collared tigers in Sariska with the help of radio-telemetry locations and also by the evidences of direct sightings and known pugmark patterns during May 2011 and January 2013. We also recorded scat location using GPS, time, date and identity of the tiger. We collected information on presence/absence of kill with every scat deposit and also the type of the kill.

We estimated the distance travelled by a tiger 72 hours prior to defecation of every scat using homing in and triangulated GPS locations and locations obtained from satellite transmitters. We calculated the Euclidean distance to the nearest waterhole, road, temple and village from all the location points, 72 hours prior to each scat deposition of individual tiger. We tried to record all the kills made by tiger in Sariska and included them in the analysis.

These fresh scat samples were thereafter transferred directly into 50 ml conical tubes (Tarson, India) containing 80% ethanol as no cold storage or lab facility was available in the field station. Prior to collection, the scat samples were mixed thoroughly using sterilized disposable plastic spoon and were disposed off after usage to avoid cross contamination. The collected and preserved samples were sent to the laboratory for analysis within a week for further extraction of steroid metabolites and hormone assays. The ethanol stored samples were further extracted within a week to avoid variation in steroid metabolite concentration due to storage as previously reported [44]. To examine variation in fecal hormone metabolite concentrations due to storage in ethanol, we analyzed hormone concentrations with reference to the number of days stored in ethanol [35], and did not find any significant differences in fecal steroid metabolite concentrations between samples stored directly at -30°C and those stored in ethanol for up to 7 days at room temperature (*p* = 0.09). Similarly, we did not find any significant difference in fecal hormone metabolite concentration post defecation for samples stored at RT for up to 24hrs in a controlled environment (*p* = 0.10).

### Assessment of anthropogenic disturbance

We divided STR into 4 km^2^ grid network to assess systematic sampling procedure. We assessed anthropogenic disturbance variables such as presence of livestock, signs of wood cutting and lopping, distances to roads, vehicular movements and presence of villagers from centre of the 4 km^2^ grids. We used 15 m radius circular plots on every 200 m distance of 52 line transects (*n* = 520), 112 radial line transect of length 2 km around 28 villages (*n* = 1148) and also from the kill, scat and homing-in locations of the reintroduced tigers (*n* = 1470). Livestock and human mean encounter rates were estimated in all line transects with nine replicate walks (three walks in three seasons across the year—summer, monsoon and winter). The individual values recorded in these 3138 plots were interpolated to develop a raster layer of human disturbance for the whole Tiger Reserve. Data on the vehicular movements on the roads going through the Tiger Reserve was collected from the forest department. We summated all these disturbance parameters to map the disturbance categories using 4 km^2^ grids and labeled the areas with different disturbance indicator categories summarizing all the values viz. low (1% to 25%), medium (25.01% to 50.00%) and high disturbance (>50.00%) of the composite disturbance range values. Further, these grids were also used for analyzing the percentage of time spent by individual tigers and their respective fGCM concentration. These disturbance categories were plotted on a multispectral high resolution (28.5 m) Landsat7 ETM+ satellite imagery (1:50,000; www.glcf.umiacs. umd.edu/ data/ landsat) of the study area which was supervised with ground truthing for better representation. Locations recorded for each tiger using both homing in and triangulation techniques (with error polygon of perimeter ranging 9m to12m for each triangulated location) were classified to the habitat type in which they occurred, to obtain habitat use. The values recorded at each point were interpolated to raster layer in the GIS domain using ArcGIS 9.3 software (ESRI, Redlands, CA, USA) and all the individual layers were then superimposed on each other over a 4 km^2^ gridded map of the entire Tiger Reserve.

### Extraction of fecal glucocorticoid metabolites

Hormone extraction was carried out using earlier described procedures [45,46]. Between 0.2–0.3 g of dried, mixed and pulverized feces were boiled in 5 ml of 90% aqueous ethanol for 20 min. After centrifugation at 500 g for 10 min, the supernatant was recovered and the pellet re-suspended in 5 ml of 90% aqueous ethanol, vortexed for 1 min and re-centrifuged to recover the supernatant. Supernatants were combined, dried (in an oven at 40°C), re-suspended in 1 ml of absolute methanol, vortexed for 1 min and sonicated for 30 s (Branson Ultrasonics 250, CT, USA), and stored at -20°C. Extraction efficiency was determined by adding a known amount of ³H labelled cortisol in fecal sample, prior to extraction and ranged between 85 to 90%.

### EIA procedure and validation

Fecal glucocorticoid metabolite (fGCM) concentrations were determined using a cortisol EIA (R4866, Dr. Coralie Munro, University of California, Davis), previously shown to provide reliable information on adrenocortical function in various mammals, including tigers [35, 47–49]. The assay was carried out as previously described by [35].

Parallel displacement curves were made between the pooled fecal extracts of tigers (endogenous) and standard (exogenous) to know the optimum fecal sample dilution (1:32) at 50% binding and immunological activity of endogenous antigen with the antibody used (S1 Fig). Assay sensitivity was calculated at 90% binding. The cortisol antibody sensitivity was found 0.002ng/g dry weight. Recovery of known amount of unlabelled exogenous cortisol was 94.63 ± 8.50 in fecal extracts analyzed by EIA. The correlation (*r* ^*2*^) and slope (*m*) values for the recovery of exogenous cortisol was *r* ^*2*^ = 0.99, *m* = 0.95. The intra- and inter-assay coefficient of variation (CV) were 8.32 and 6.3 (*n* = 10) for low and 8.87 and 7.62 (*n* = 10) for high binding controls.

### Statistical analysis

Non-parametric correlation analysis was performed between fGCM and various disturbance parameters (wood cutting, lopping, livestock encounter rate, and distance to major roads, villages and water holes). The difference between mean cortisol values of individual tigers and anthropogenic variables were analyzed using Student’s t-test. One-way ANOVA was used to compare variation of fGCM concentrations with reference to individuals, months and seasons. General linear model (GLM) procedures with repeated measures, was used to evaluate changes in hormone concentrations between sexes. Further, generalized linear model (GzLM) was used to determine causes of variation of fGCM concentration with reference to various anthropogenic factors as the explanatory variables that included both continuous and categorical data and the response variable was continuous data. Prior to modeling, all the continuous data were subjected to correlation analysis to test for autocorrelation amongst the explanatory variables. It was found that encounter rate of livestock and human have strong positive correlation, accordingly, the counter rate of human was excluded in the final model. GzLM was built with Poisson distribution with identity link function; given that the dependent variables contain non-normal distribution data and that the variables were not required to be transformed. The model was performed with main effects function so as to understand the exact dimension of the effects caused by the individual variables. To avoid pseudoreplication, we used individual ID as fixed effect in the GLM. Parameter estimation was performed following hybrid method with maximum iterations of 100. Bivariate non-parametric correlation between fGCM concentration and time since reintroduction was also done to understand the effect of translocation stress. The parameter estimation was done with 95% confidence interval. All the analyses were carried out using SPSS ver 17.1.

## Results

Tigers in Sariska were observed to move on an average 20.58 ± 0.86 km (mean ± SE; range = 1.4–47.9 km) in 72 hours before scat sample deposition. Average movement of tigers and fGCM concentrations were not correlated (*r*_*s*_ = 0.09; *p* = 0.32; *n* = 120). Further, no sex-related difference was found when comparing average animal movement in males (22.19 ± 1.27 km) and females (19.57 ± 1.14 km; *p* = 0.23). Tigers were found staying away from human settlements for an average 2.50 ± 0.76 km and maintained a distance of at least 0.66 ± 0.11 km from the nearest water hole.

In total 120 tiger scat samples were collected from 5 individuals (mean = 24 samples; range = 13–32 samples) in STR for fGCM analysis. Overall individual fGCM concentrations varied significantly during the study period (*F*_*4*_,_*119*_ = 2.97, *p < 0*.*05* with female tiger ST2 showing the highest individual mean fGCM values (115.68 ± 24.62 ng/g) followed by females ST5 (83.46 ± 18.46 ng/g) and ST3 (66.12 ± 56.83 ng/g) In contrast, male ST6 showed the lowest individual mean fGCM values (47.90 ± 7.75 ng/g). Samples from female tigers (89.56 ± 11.63 ng/g, *n* = 71) showed significantly higher fGCM concentrations than respective samples from males (54.13 ± 6.75 ng/g, *n* = 49; GLM Wald χ^2^ = 529.47, *p* < 0.001). No significant differences in fGCM concentrations were found between months (*F*_*4*_,_*11*_ = 0.91, *p* > 0.05 and seasons (*F*_*4*,*3*_ = 0.97, *p* > 0.05 such as summer (March—June), rainy (July—October) and winter (November- February).

**Fig 2:**
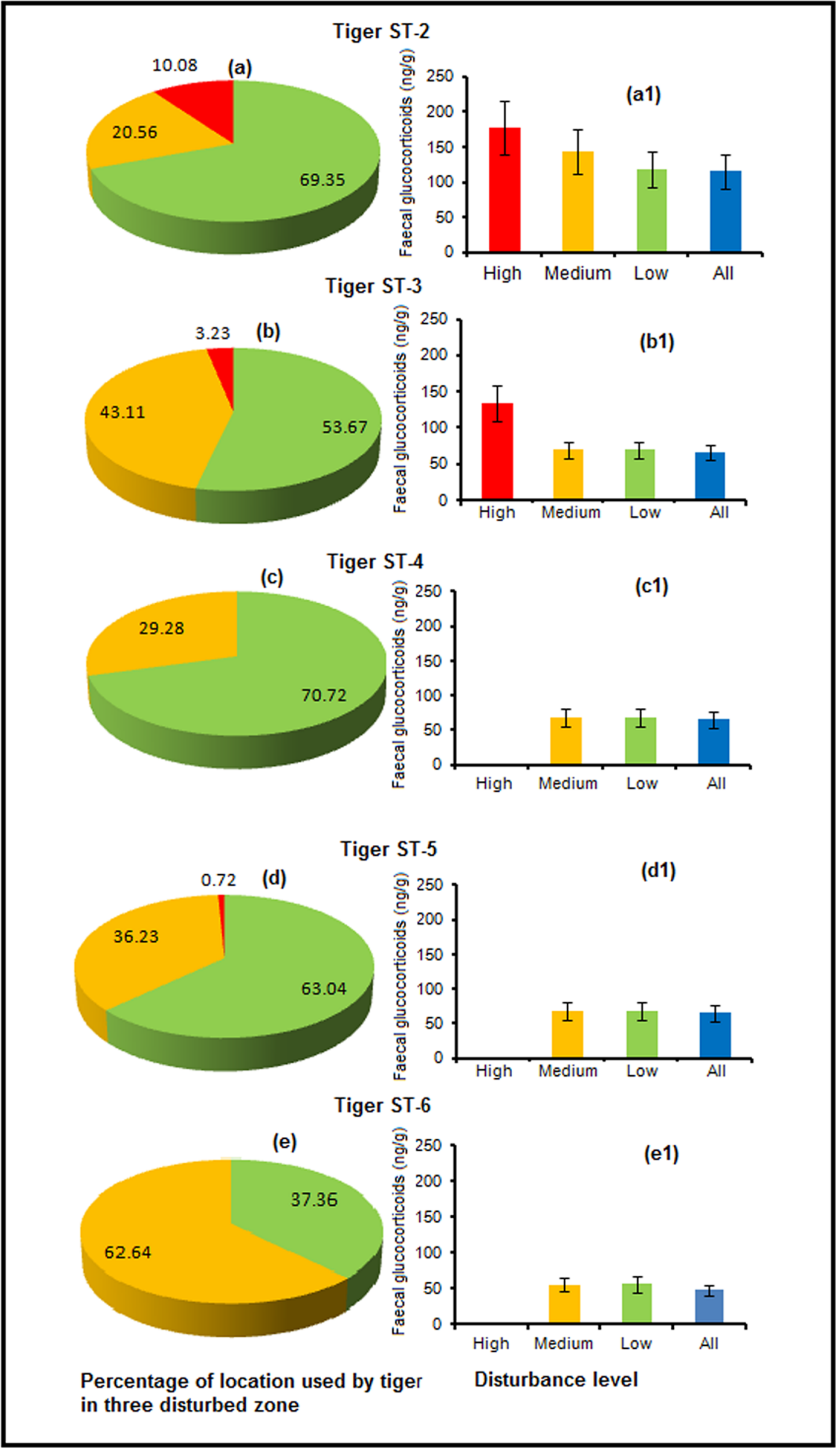
Percentage of radio location of tigers in different disturbed areas and respective (subset figures) mean faecal glucocorticoid metabolite concentration in Sariska Tiger Reserve. (Red- high disturbance, yellow—medium disturbance, green—less disturbance zones and blue—overall mean +/- SE).

Fecal GCM levels were found to be significantly correlated to various evaluated anthropogenic variables (Table 1). Of these, the encounter rate with livestock, predation events of domestic animals (such as cattle and buffaloes), and distance to roads were identified to distinctively contribute to elevated fGCM concentrations (Table 1). Further, we found that fGCM levels of tigers are higher when the animals have been located in areas where human and cattle presence were common (human encounter rate vs fGCM, *r*_*s*_ = 0.50; *p* = 0.001, *n* = 120; livestock encounter rate vs fGCM, *r*_*s*_ = 0.61, *p* = 0.001, *n* = 120). Fecal GCM levels linked to tigers preying on domestic livestock were also higher (112.09 ± 43.69ng/g) compared to when preying on wildlife (41.25± 12.09). Interestingly, most of the domestic livestock kills were recorded in the disturbed areas (24 out of 26) within 1.98 ± 0.30 km from the roads and 2.56 ± 0.13 km from the villages. Overall, fGCM concentrations were found to increase with increasing time spent in disturbed areas (*r*_*s*_ = 0.57; *p* = 0.04, Fig 2 and S2–S6 Figs). Tigress ST2, in particular, which spent about 10.30% of its time in the high disturbance zones (all other tigers spend between 0–3.2% in those zones), showed an overall increase of 67% in fGCM concentrations during this time compared to respective fGCM values revealed when the animals was located in low disturbance zones. Further, fGCM values were not affected by the duration since translocation to Sariska (*r*_*s*_ = -0.04, *p* = 0.66, *n* = 120) suggesting that the fGCM concentrations were not influenced by translocation stress.

**Table 1:** Generalized linear model (with Poisson distribution and identity function) of factors that influenced fecal glucocorticoid metabolite concentrations in tigers of Sariska Tiger Reserve (2011–2013).

Parameter	B*	Std. Error	B Interval		Wald Chi-Square	df^#^	Sig^$^
Intercept	54.04	3.53	47.11	60.96	233.83	1	.000
Sex	-23.30	1.52	-26.28	-20.32	234.93	1	.000
Livestock kill	-69.55	4.3	-78.02	-61.07	258.61	1	.000
Mean distance road	-13.32	0.6	-14.60	-12.04	415.93	1	.000
Encounter rate with livestock	26.76	0.64	25.51	28.01	1764.75	1	.000
Total movement prior to defaecation	0.56	0.07	0.42	0.70	61.52	1	.000
Mean distance to village	0.68	0.89	-1.07	2.41	0.58	1	.447
Mean distance to water hole	-13.43	4.13	-21.53	-5.33	10.57	1	.001

*—Beta or Coefficient

^#-^ Degrees of Freedom

^$^Sig. is Significant level at 95% Confidence Interval

## Discussion

The study revealed that the tigers reintroduced in Sariska are affected by anthropogenic disturbances causing an enhanced stress response. Although we cannot exclude the possibility of sex [50] or potential body size-related differences in fGCM output, it seems that female tigers are more susceptible to the monitored anthropogenic disturbances than males, as fGCM elevation appeared more distinctively in females during domestic livestock kills, moving in disturbed areas and while encountering humans and livestock. A recent study on African lions demonstrated that lions roaming within a human-settled buffer zone show higher fGCM concentrations compared to lions living in conservation areas [32]. Further, fGCM concentrations also decreased with increasing distance to human settlement in these animals [32]. Similarly, a study conducted on spotted hyenas (*Crocuta crocuta*) in the Masai Mara National Reserve, Kenya, demonstrated that animals in disturbed areas had significantly higher fGCM concentrations than those in undisturbed areas [30]. A similar observation was made in this study, where tiger movement, particularly in areas with a high number of livestock, vehicular traffic and presence of human settlements, seem to be associated with an increase in fGCM concentrations in the tigers in Sariska. Unfortunately, most of the Sariska Tiger Reserve areas had to be categorized as highly disturbed areas due to the frequent heavy vehicular traffic, the presence of human settlements and livestock, and wood cutting and lopping (S2–S6 Figs.). One possible explanation for the higher fGCM concentrations might be that these tigers encounter very high vehicular traffic, herders men, villagers who frequently visit the forests for fuel wood collection and livestock grazing. This is aggravated by the fact that most of the natural water holes dry up during the dry season and artificial water tanks, being the dominant water source for wild animals during this time, were mostly built near to the state highways and major roads for easy refilling by mobile vehicles. For example, these water tanks were regularly used by the two tigresses (ST2 and ST3) showing highest individual mean fGCM concentrations. In contrast, male ST-6 which had a large home range which also includes some of the disturbed areas, showed comparatively very low fGCM concentrations throughout the year. Interestingly, this individual was brought to Sariska from Keoladeo National Park Bharatpur, Rajasthan, and spent about 5–6 months in a human dominated landscape before it was chemically immobilized, radio-collared and released into STR. It could be speculated that his preceding experiences were subsequently beneficial to overcome various anthropogenic disturbances after reintroduction in Sariska.

Prolonged stress has the potential to negatively influence reproduction by acting on the hypothalamus, pituitary gland, or the gonads [33]. Studies showed that glucocorticoids can inhibit gonadotropin secretion in some circumstances and are likely to be influenced by sex of the individual [51]. In the present study, behavioral estrus was observed in all the female tigers (data not shown) on a regular basis and observed with courtships. However, ST2 littered two cubs in the month of June [21] after four years of re-introduction when she was finally able to roam in a least disturbed area of Slopka to Kundli range. Our field data further shows that this female has stayed in these areas during conception and the gestation period, indicating that less stressful environmental conditions might support reproductive success. In contrast, three of the five reintroduced tigresses in Panna Tiger Reserve produced multiple litters successfully over these years and the reason for the same was attributed to very less anthropogenic activities in their established home ranges [20]. One of the reasons for the overall low breeding success in Sariska might therefore be the prolonged elevation in fGCM levels among the female tigers observed throughout the study period. As mentioned above, it took nearly four years for the ST2 tigress to find an undisturbed 3 km^2^ area (Slopka to Kundli), within her home range to successfully litter and raise her cubs. The other tigresses, ST3 and ST5 did not produce any litter till the completion of our study, though there were observed to be periodically mating with the male tigers.

Management recommendations include regulation of vehicular traffic, shifting of artificial water holes away from tarmac roads and relocation of eight villages from the core area of the Tiger Reserve which are due for relocation since 1984. Once these villages are relocated, an inviolate area of around 300 km^2^ will be available for tiger breeding. A combined approach of behavioral observations with non-invasive longitudinal hormone measurements will elucidate the endocrine milieu underlying reproductive behavior in these apex predators, opening new opportunities to improve management and welfare of the tigers in STR, thereby assisting conservation efforts.

## Supporting Information

S1 Fig Parallelism between pooled serial dilution of tiger’s fecal extract (square) and respective cortisol standard (circle). (TIF)

S2 Fig Different levels of anthropogenic disturbance and ST2 tigress movements prior to scat deposition during the study period in Sariska Tiger Reserve (May 2011—January 2013). (TIF)

S3 Fig Different levels of anthropogenic disturbance and ST3 tigress movements prior to scat deposition during the study period in Sariska Tiger Reserve (May 2011—January 2013). (TIF)

S4 Fig Different levels of anthropogenic disturbance and ST4 tiger movements prior to scat deposition during the study period in Sariska Tiger Reserve (May 2011—January 2013). (TIF)

S5 Fig Different levels of anthropogenic disturbance and ST5 tigress movements prior to scat deposition during the study period in Sariska Tiger Reserve (May 2011—January 2013). (TIF)

S6 Fig Different levels of anthropogenic disturbance and ST6 tiger movements prior to scat deposition during the study period in Sariska Tiger Reserve (May 2011—January 2013). (TIF)
