# Supplementary figures and images for: Glucocorticoid Stress Responses of Reintroduced Tigers in Relation to Anthropogenic Disturbance in Sariska Tiger Reserve in India
Source: PLoS One. 2015 Jun 10;10(6):e0127626. doi: 10.1371/journal.pone.0127626 (PMC4465644; doi:10.1371/journal.pone.0127626)

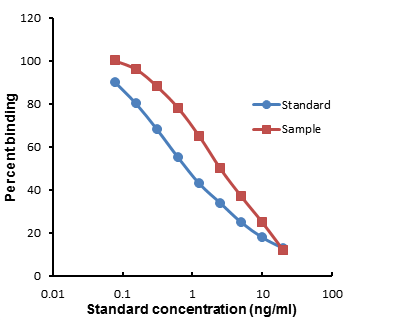

Supplement: S1 Fig — (TIF) [file pone.0127626.s001.tif]

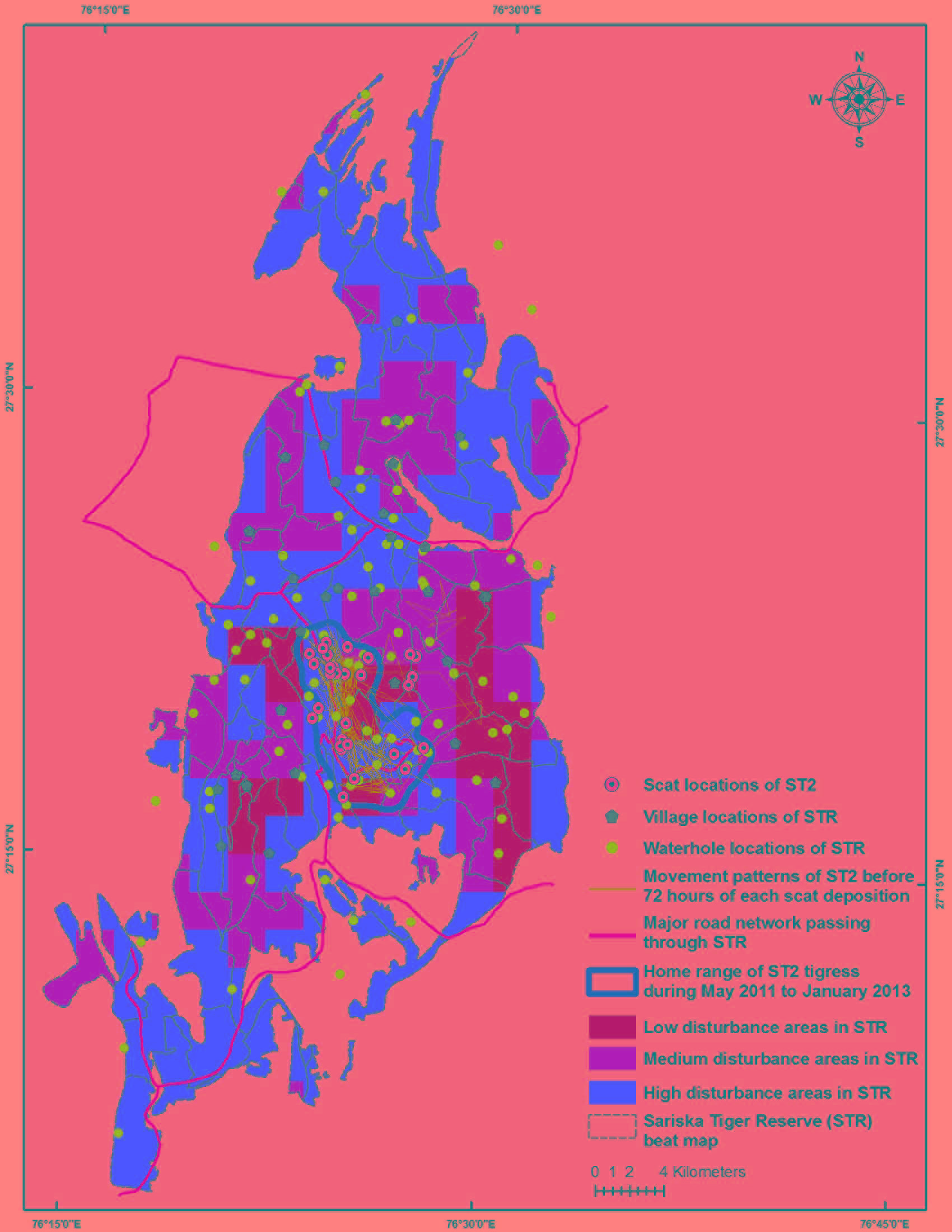

Supplement: S2 Fig — (TIF) [file pone.0127626.s002.tif]

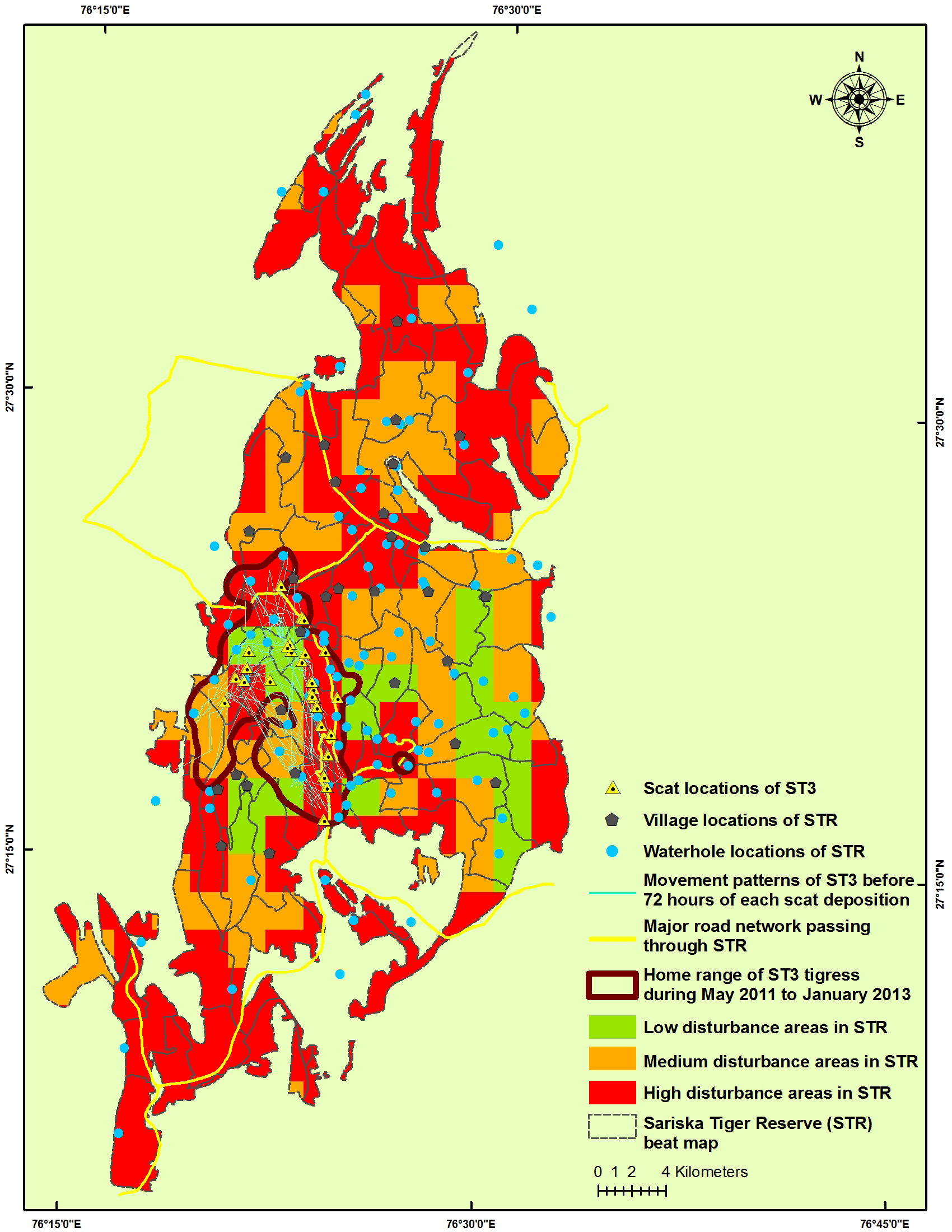

Supplement: S3 Fig — (TIF) [file pone.0127626.s003.tif]

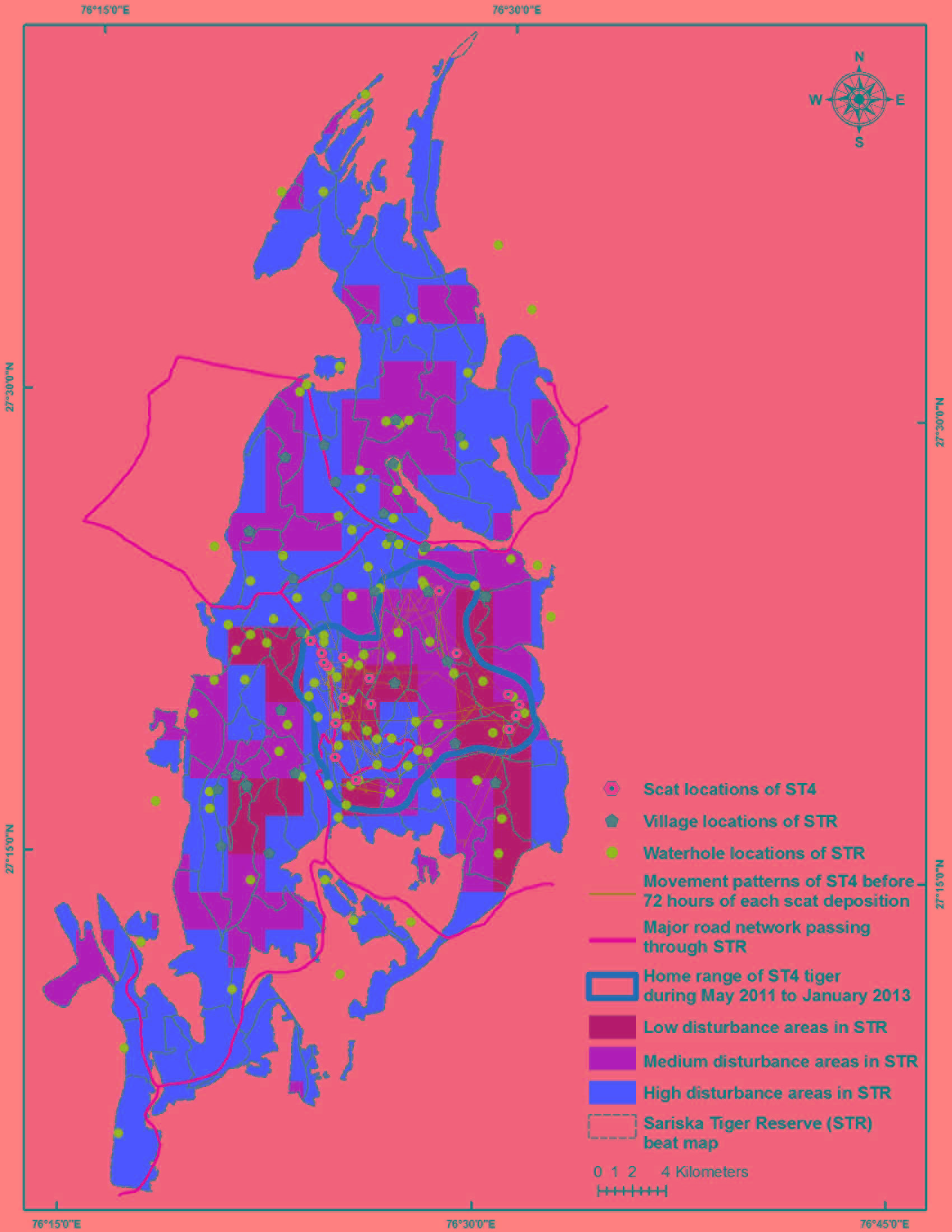

Supplement: S4 Fig — (TIF) [file pone.0127626.s004.tif]

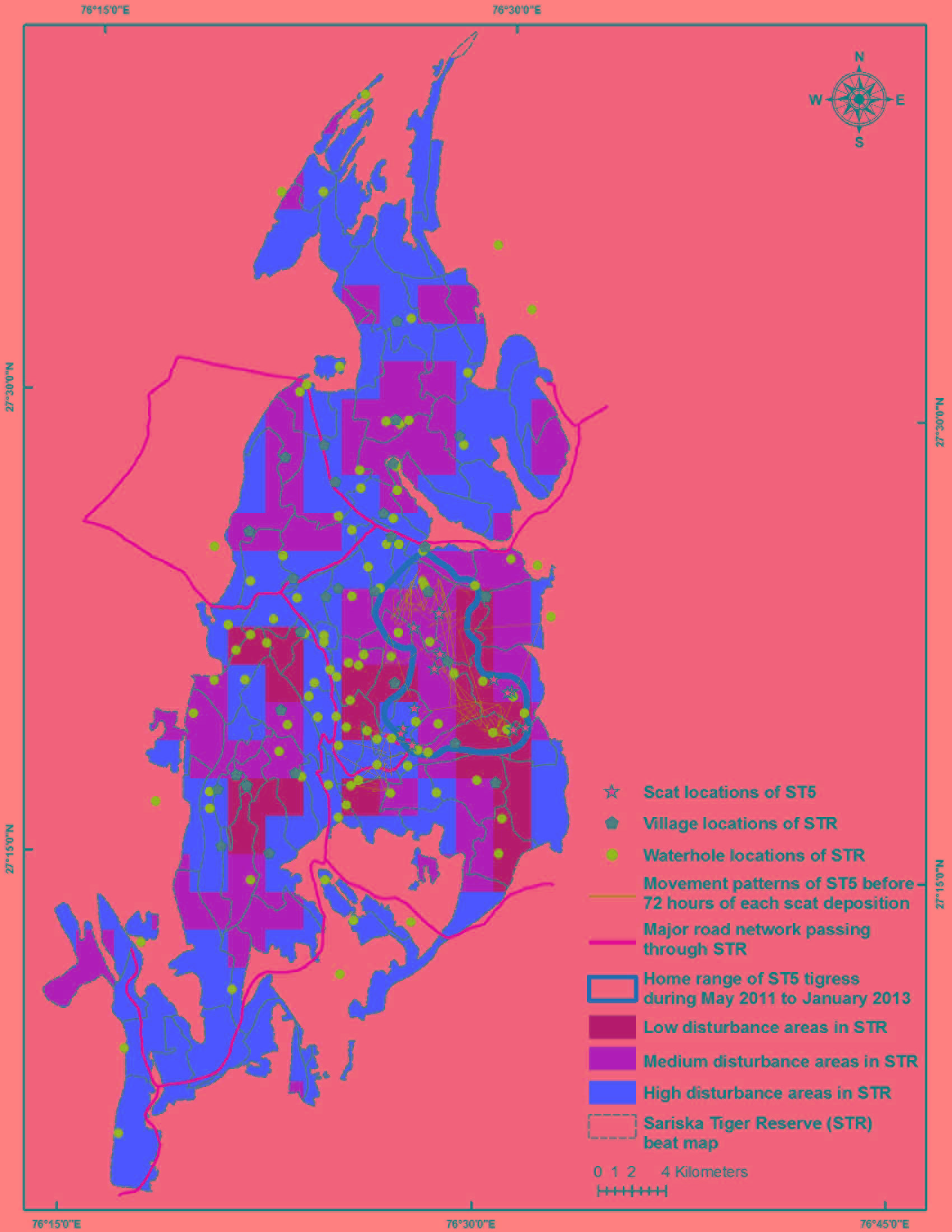

Supplement: S5 Fig — (TIF) [file pone.0127626.s005.tif]

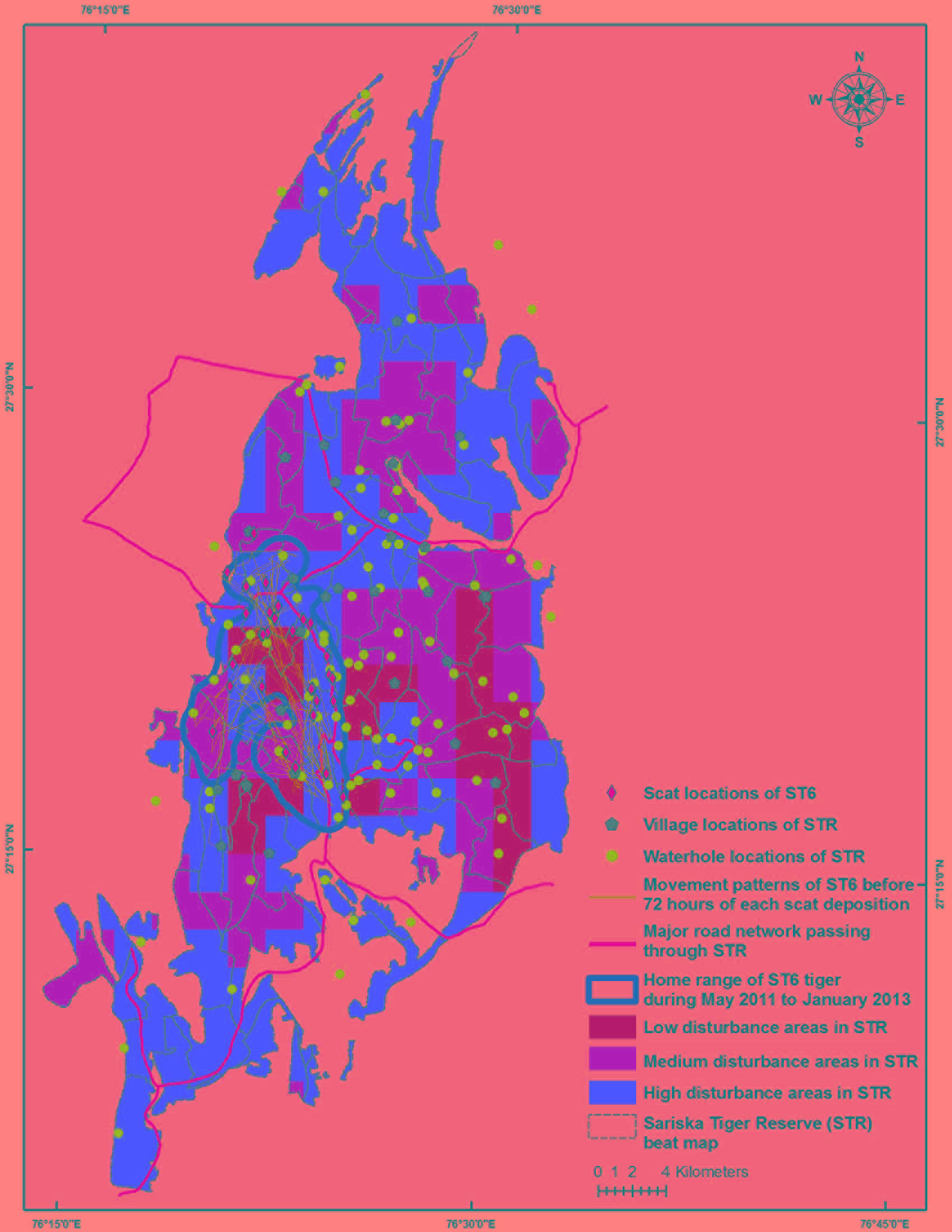

Supplement: S6 Fig — (TIF) [file pone.0127626.s006.tif]
